# Exosomes from normal and diabetic human corneolimbal keratocytes differentially regulate migration, proliferation and marker expression of limbal epithelial cells

**DOI:** 10.1038/s41598-018-33169-5

**Published:** 2018-10-11

**Authors:** Aleksandra Leszczynska, Mangesh Kulkarni, Alexander V. Ljubimov, Mehrnoosh Saghizadeh

**Affiliations:** 1 Biomedical Sciences, Cedars-Sinai Medical Center, Los Angeles, California USA; 2 Regenerative Medicine Institute Eye Program, Cedars-Sinai Medical Center, Los Angeles, California USA; 3 David Geffen School of Medicine, University of California Los Angeles, Los Angeles, California USA

## Abstract

Limbal epithelial stem cells (LESC) maintenance requires communication between stem cells and neighboring stromal keratocytes. Extracellular vesicles (EVs) are important for intercellular communication in various stem cell niches. We explored the regulatory roles of limbal stromal cell (LSC)-derived exosomes (Exos), an EV sub-population, in limbal epithelial cells (LEC) in normal and diabetic limbal niche and determined differences in Exo cargos from normal and diabetic LSC. Wound healing and proliferation rates in primary normal LEC were significantly enhanced upon treatment by normal Exos (N-Exos), but not by diabetic Exos (DM-Exos). Western analysis showed increased Akt phosphorylation in wounded LECs and organ-cultured corneas treated with N-Exos, compared to untreated wounded cells and DM-Exos treated fellow corneas, respectively. N-Exos treated organ-cultured corneas showed upregulation of putative LESC markers, keratin 15 (K15) and Frizzled-7, compared to the DM-Exos treated fellow corneas. By next generation sequencing, we identified differentially expressed small RNAs including microRNAs in DM-Exos *vs*. N-Exos. Overall, N-Exos have greater effect on LEC proliferation and wound healing than DM-Exos, likely by activating Akt signaling. The small RNA differences in Exos from diabetic *vs*. normal LSC could contribute to the disease state. Our study suggests that exosomes may serve as novel therapeutic tools for diabetic cornea.

## Introduction

The homeostasis of corneal epithelium is a dynamic and complex process that plays a key role in the corneal transparency and visual function. Renewal of terminally differentiated central corneal epithelium, essential of transparency, is orchestrated through the differentiation and centripetal migration by the limbal epithelial stem cells (LESC). Thus, maintenance of LESC in their niche environment is critical for proper functioning of corneal epithelium. LESC are quiescent cells located at the corneal periphery in the corneoscleral limbus inside specific structures called palisades of Vogt^1^, and/or in the deeper limbal epithelial crypts and focal stromal projections^2–4^. Unlike central epithelial cells, LESC are in close contact with the cells in the underlying limbal stroma and the vasculature that surrounds the limbal crypts^5^.

It is well established that limbal stromal cells (LSC), or keratocytes, support limbal epithelial cell (LEC) homeostasis through their secreted soluble factors^6–9^. Central corneal stroma promotes cell proliferation and differentiation, whereas limbal stroma helps maintaining cell stemness^10^. Therefore, the stem cell maintenance and function in normal and diseased states of the cornea involve complex interactions of various intrinsic and extrinsic factors between all the resident cell types as well as infiltrating cells from the circulation in the limbal stromal microenvironment, or niche. Any damage to LESC or limbal stromal niche due to the external insults or diseases such as diabetes may lead to pathological state of altered vision, and in severe cases of LESC loss may lead to limbal stem cell deficiency (LSCD) and blindness.

Recent studies have shown the important roles of extracellular vesicles (EVs), in addition to direct cell-cell contact or secreted molecules, in communication between the surrounding cells and ECM in stem cell maintenance and activation^11^. There are different types of EVs including exosomes, microvesicles, ectosomes or shedding vesicles, which differ by their subcellular origin, type of release, and size, and are secreted by most cells and contain mRNA, microRNA, DNA and protein cargo mediating physiological intercellular crosstalk^12^. An EV sub-population, exosomes (Exos), are small endosomal membrane-bound vesicles about 50–200 nm, with a range of nucleic acids and proteins contents, which differ with the cell and tissue of origin^12^. They exert their effects by fusion to the target cells and transferring their cargo, which may include bioactive molecules such as proteins, lipids, mRNAs and miRNAs^12^. The important roles of exosomes have been shown in pathological conditions, such as cancer^13^, inflammation^14^, cardiovascular diseases^15,16^, diabetes^17,18^, as well as in wound healing^19,20^. Exosome-like vesicles were described between central corneal epithelial cells and the stroma during wound healing after epithelial debridement of mouse cornea^21^.

In this study, we characterized both healthy or normal (N) and diabetic (DM) human limbal LSC-derived Exos and examined their roles in survival, migration and proliferation of LEC in normal and diabetic corneas. Furthermore, next generation sequencing (NGS) was performed to identify distinct miRNA players and investigate the effect of diabetes on LSC-derived Exo population. Our study indicates that normal LSC-derived Exos (N-Exos) have a greater potential in cell proliferation and wound healing than diabetic LSC-derived Exos (DM-Exos). Using NGS analysis, we have also documented differences in Exo cargos derived from normal and diabetic limbal keratocytes.

## Results

### Primary limbal stromal cells (keratocytes) characterization

Corneal stromal cells isolated from the limbal region were characterized based on their morphology and specific marker expression. The morphology was typical of LSC^22,23^, that is, elongated, spindle-shaped with long spreading cellular processes, which was maintained at confluence and at later passages (Supplementary Fig. S1a). Immunocytochemistry showed positive staining for lumican, keratocan and aldehyde dehydrogenase 3 (ALDH3), which are specific keratocyte markers (Supplementary Fig. S1b). The staining for myofibroblast marker α-SMA, and corneal epithelial marker keratin 12 (K12) was negative (Supplementary Fig. S1b). Flow cytometry further confirmed the expression of lumican and ALDH3 (Supplementary Fig. S1c). Western blot analysis also showed lumican expression in both normal and diabetic keratocytes (Supplementary Fig. S1d).

### Characterization of normal and diabetic human limbal LSC-derived Exos

**Figure 1:**
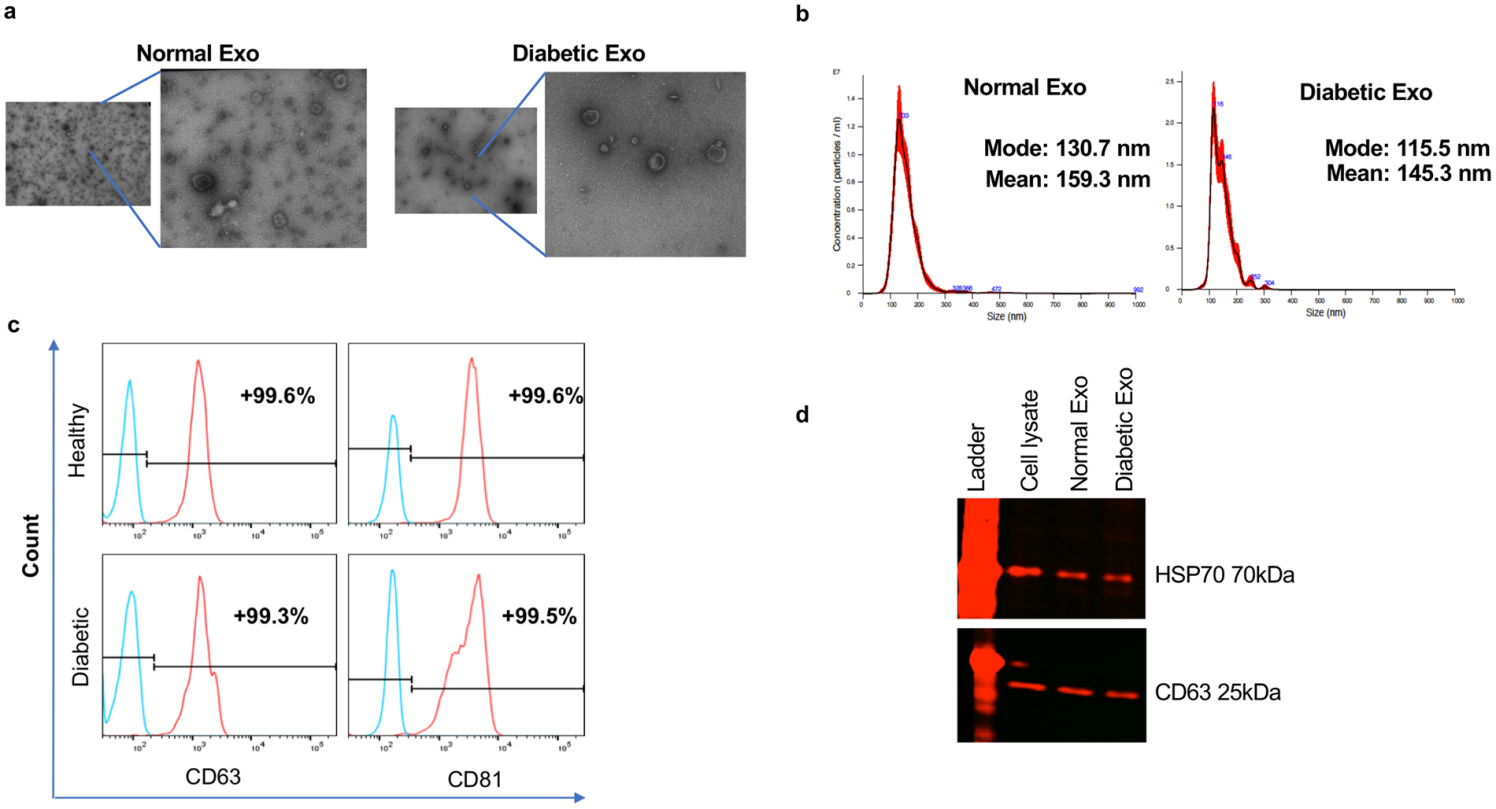
Characterization of normal and DM human LSC-derived Exos. (**a**) Representative TEM images showing a range of exosomal size from 50–200 nm and typical doughnut shape morphology in both N (n = 3) and DM (n = 3) human LSC-derived Exos. (**b**) size distribution of LSC-derived EVs was determined by NanoSight LM10. Histogram shows particle size distribution typical of exosomes. (**c**) Flow cytometry was performed on normal (healthy)- and DM-EVs using anti-CD63-coated beads. Vesicles were immunostained against CD63 (red) and CD81 (red) and compared with appropriate isotype control (blue), n = 3. (**d**) Western blot showed expression of typical exosomal markers HSP70 and CD63 in both N (n = 3) and DM (n = 3) vesicles.

Exos derived from conditioned media of at least three biological replicates of each normal and diabetic keratocytes were characterized by several analytic approaches. The typical cup shape EV morphology was detected by transmission electron microscopy (TEM, Fig. 1a). EV size was measured using TEM and NanoSight technology (Fig. 1a,b) with the size ranging between 50 and 200 nm (mean size 159.3 nm for normal EVs and 145.3 nm for diabetic EVs). We used common exosome markers to characterize normal and diabetic human LSC-derived EVs by flow cytometry and western blotting (Fig. 1c,d). Flow cytometry using BD LSR II instrument (BD Biosciences, San Jose, CA), showed that both normal and diabetic EVs were positive for both CD63 and CD81, with no significant difference (using Student’s *t*-Test, n = 3, p > 0.05) between two groups (Fig. 1c). Western analysis of EVs showed positive bands for CD63 and heat-shock protein (HSP)70 (Fig. 1d). The data suggested that the majority of our isolated EVs were exosomes.

### Internalization of Exos by cultured cells and organ-cultured corneas

**Figure 2:**
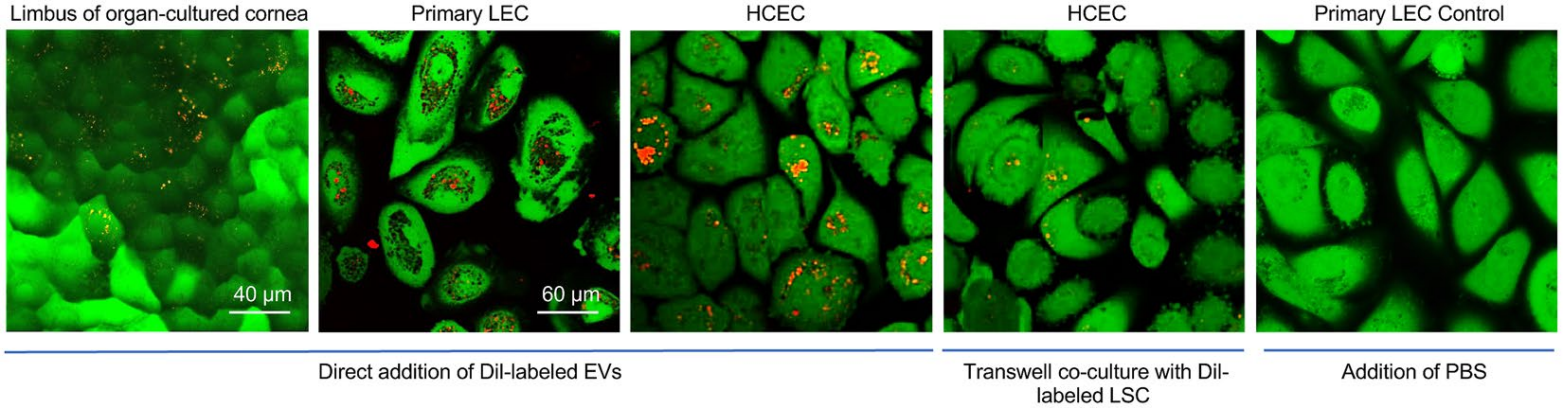
Dil-labeled normal human LSC-derived EVs can be internalized by human primary LEC, HCEC and organ-cultured corneas. Dil-labeled LSC-derived Exos (25 μg/ml or 3 × 10^8^/ml) were incubated (direct addition of Dil-labeled Exos) with organ-cultured corneas, primary LEC and HCEC for 24 h, no Exo was added to the control. Dil-labeled normal keratocytes were co-cultured up to 48 h on trans-wells. The cells were stained with calcein-AM, which exhibits green fluorescence and demonstrates live cells and their uptake of EVs.

To determine whether human limbal epithelial cells are the targets for LSC-derived Exos, Dil red fluorescent dye was used to label Exos. Primary LEC were treated with 10 and 25 μg/ml Dil-labeled LSC-derived Exos for 24 h to optimize the internalization of Exos by cultured cells (Supplementary Fig. S2). After incubating the labeled Exos (25 μg/ml or 3 × 10^8^/ml; this dose was chosen for all other experiments) with organ-cultured corneas, primary LEC and telomerase immortalized human corneal epithelial cells (HCEC) for 24 h, the uptaken Exos were observed in limbal region of organ-cultured corneas (Fig. 2) and in perinuclear region of both cell types, LEC and HCEC, by confocal microscopy (Fig. 2), demonstrating the internalization of labeled Exos by live cells. Similar results were observed in co-culture system where Dil-labeled human LSC were seeded onto the 0.4 µm inserts in transwell system with HCEC as a recipient (Fig. 2).

### Exos derived from normal but not DM LSC enhance cell proliferation and wound healing rate in normal LEC *in vitro*

**Figure 3:**
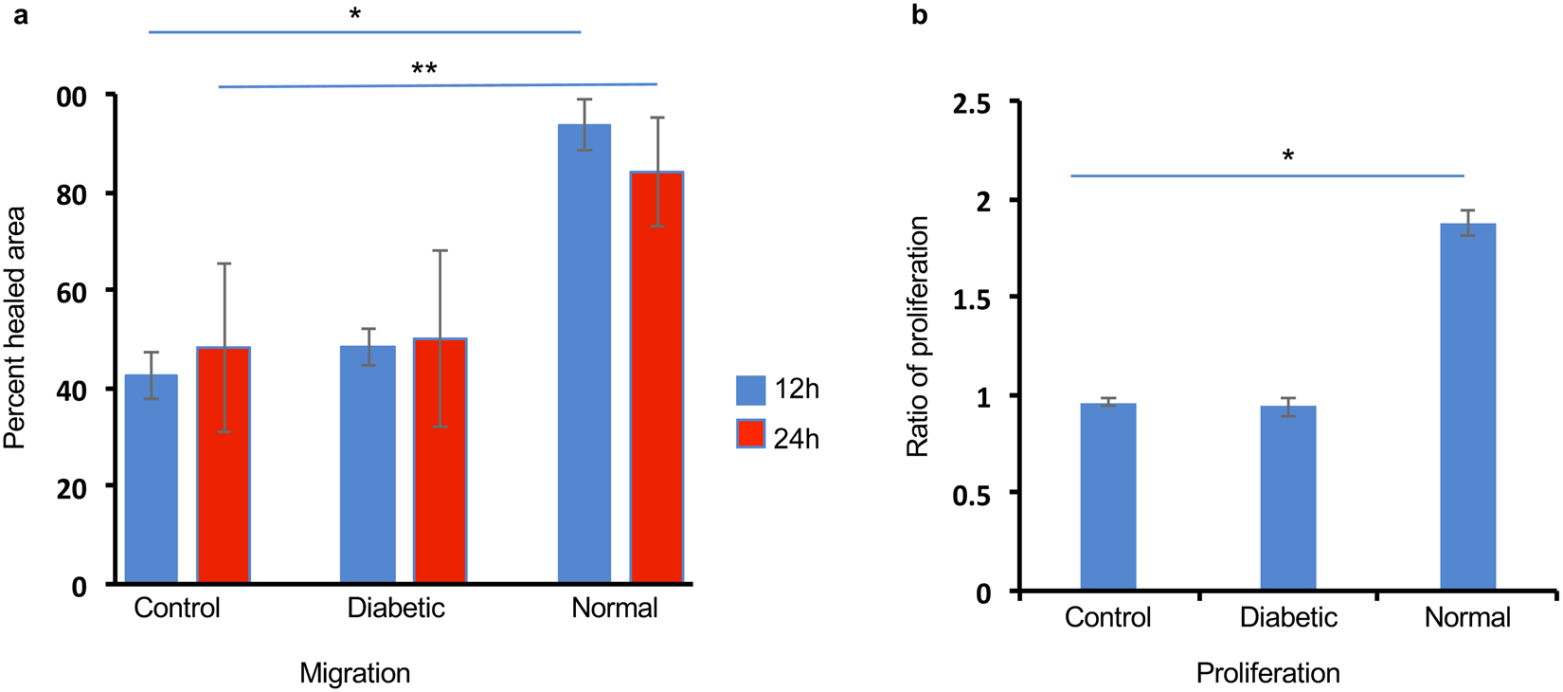
Normal EVs increase migration and proliferation of normal primary LEC. (**a**) LEC were scratch-wounded and incubated with 25 μg/ml N/DM LSC-derived EV, and wound closure was quantified using ImageJ software at 12 and 24 hr after wounding. Cell migration and wound closure were significantly enhanced in normal primary LEC treated with N-Exos compared to the cells treated with DM-Exos or PBS/untreated control cells. DM-Exos treatments did not change the wound healing rate compared to control. (**b**) MTS proliferation assay showed increase in proliferation rate in LEC treated with N-Exos *vs*. those treated with DM-Exos or untreated control cells. DM-Exo treatments did not change the proliferation rate compared to control. The bar graph represents average ± SEM of pooled values of three independent triplicate assays and compared to untreated control cells (negative control). **p < 0.01, *p < 0.05 by paired two-tailed t test.

Scratch-wounded cultured LEC treated with N-Exos increased wound healing rate compared to untreated control cells (Fig. 3a). In addition, LEC treatment with N-Exos for 24 h increased cell proliferation rate compared to untreated control cells (Fig. 3b). Interestingly, DM-Exos didn’t exert significant effects on proliferation or migration rates in treated LEC compared to control cells (Fig. 3a,b). Additionally, immunostaining of normal primary LECs treated with N-Exos showed a similar increase in Ki-67 compared to those treated with DM-Exos or untreated control cells (Supplementary Fig. S3). DM-Exo treatments decreased the expression of Ki-67 compared to untreated control cells (Supplementary Fig. S3).

### Effects of Exos on activation of wound healing-related signaling molecules in primary LEC and organ-cultured corneas

**Figure 4:**
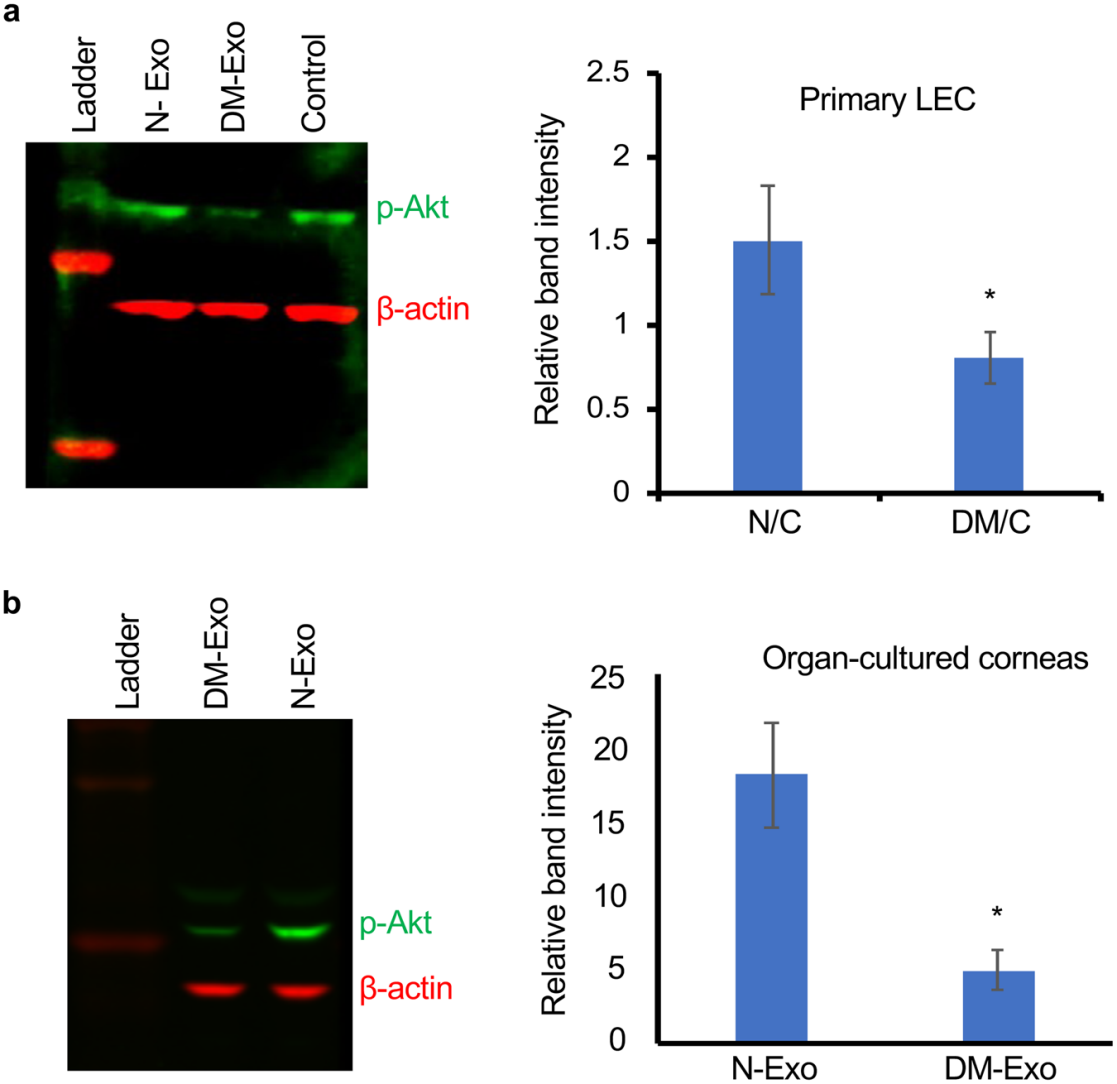
Western blot analysis of p-Akt expression in wounded LEC and organ-cultured corneas treated with normal or DM LSC-derived Exos. (**a**) Total extracted protein from wounded primary LEC treated with N/DM Exos and untreated cells (control) was separated on gradient SDS-PAGE gels, transferred to nitrocellulose and probed with antibodies to p-Akt. Normal-Exo treatment increases protein levels of p-Akt *vs*. control (PBS/untreated) and DM-Exo treated cells. (**b**) Western analysis showed increases p-Akt expression in wounded organ-cultured corneas treated with N-Exos compared to the fellow corneas treated with DM-Exos. Antibody to β-actin was used as equal loading control and for semi-quantitation. All experiments were performed in triplicate. *p < 0.05 by paired two-tailed t test.

To investigate the effect of normal and diabetic Exos on signaling pathways during wound healing, LEC cultures and organ-cultured corneas were wounded and treated with either N-Exos or DM-Exos. Western analysis of wound healing related signaling molecules showed significantly increased expression of p-Akt in wounded LEC incubated with N-Exos compared to control untreated cells, whereas DM-Exos treated wounded cells showed significantly decreased expression of p-Akt compared to control untreated cells (Fig. 4a). Similarly, wounded organ-cultured corneas incubated with N-Exos showed increased level of p-Akt protein levels in comparison with the fellow corneas incubated with DM-Exos (Fig. 4b). However, there were no significant differences in levels of p-p38 among any treatments both in LEC cultures and organ-cultured corneas (Supplementary Fig. S4).

### Effects of Exos on LESC markers

**Figure 5:**
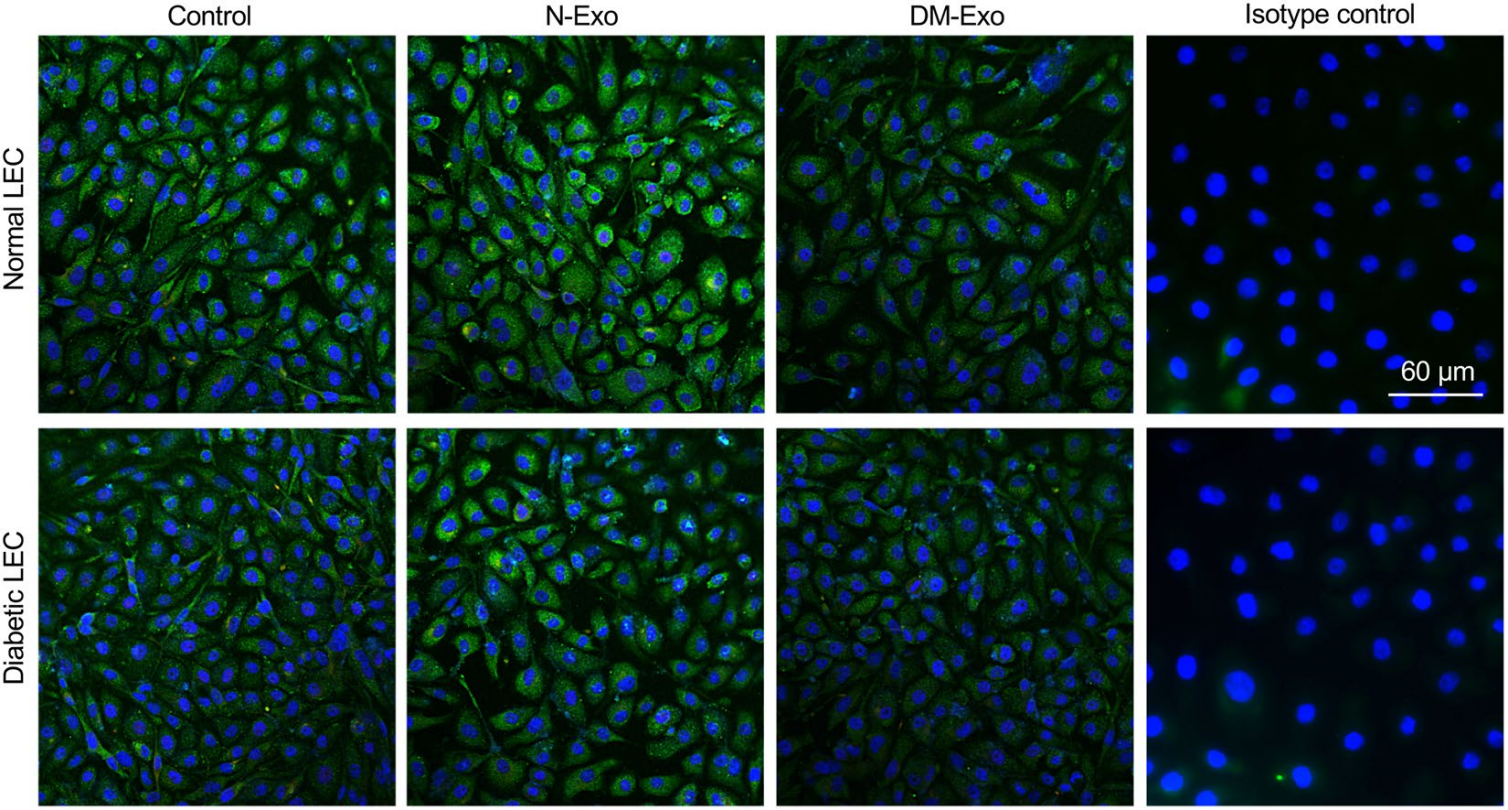
Effect of N and DM Exos on the expression of putative LESC marker, K15, in normal and DM primary LEC. Both normal and DM LEC treated with N-Exos increased K15 expression in comparison with untreated control or DM-Exo treated cells. DM-Exo treated cells showed no change in K15 expression level in comparison to control cells. IgG2aκ isotype control antibody (Thermo Fisher Scientific) was used as negative control. The experiments were performed at least in triplicate and same exposure time was used for each set of compared stained sections, and the assessment was done by more than one observer.

**Figure 6:**
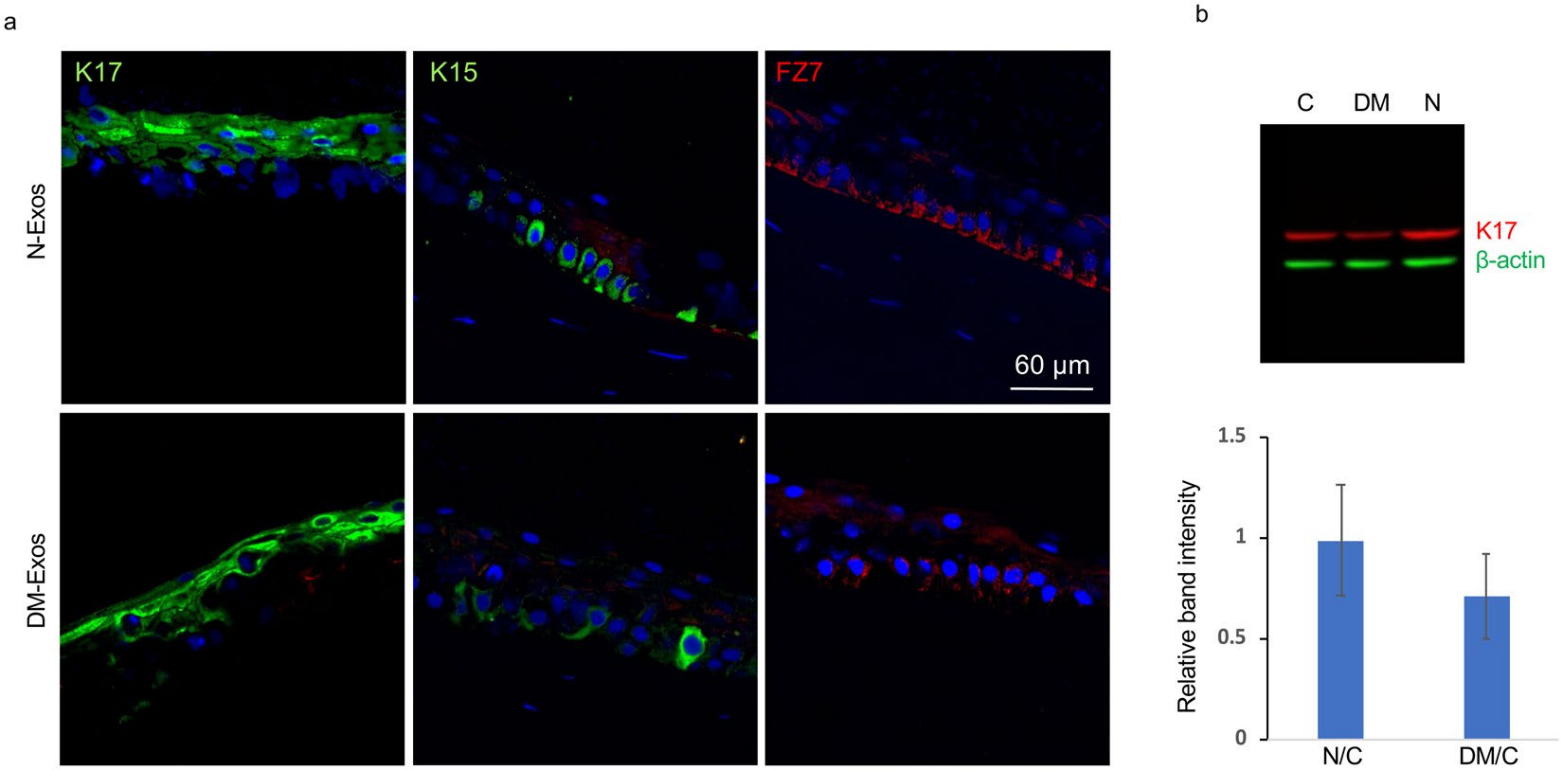
Effect of N- or DM-Exos on LESC marker expression in normal organ-cultured corneas and primary LEC. (**a**) Normal Exo treatment in normal organ-cultured corneas led to increased expression of putative LESC markers, K15 and FZ7, and no significant change in K17 protein level compared to fellow corneas treated with DM-Exos (immunofluorescent staining of limbal corneal sections). The same exposure time was used for each set of compared stained sections, and the assessment was done by more than one observer. (**b**) Western analysis shows that N-Exos treatment increased, whereas DM-Exo treatment decreased K17 protein expression level in primary LEC compared to control treated cells, which did not reach significance. Antibody to β-actin was used as equal loading control and for semi-quantitation.

The effects of N-Exos and DM-Exos on the expression of putative LESC markers were examined in primary normal LEC cultures and in organ-cultured corneas by immunostaining and western blot. Our data showed increased staining of putative stem cell marker, K15, in LEC treated with N-Exos compared to DM-Exo treated cells or untreated control LEC (Fig. 5). In addition, normal organ-cultured corneas treated with N-Exos showed upregulation, whereas corneas treated with DM-Exos showed downregulation of putative LESC markers K15, frizzled-7 (FZ7), and to some degree K17 (Fig. 6a). Western blot analysis showed N-Exo treatment increased, while the DM-Exo treatment decreased K17 protein expression level in primary LEC compared to control, which did not reach significance (Fig. 6b).

### Distinct small RNA profile of normal and diabetic LSC-derived Exos

We conducted a comprehensive analysis of small RNAs including microRNA expression in normal and diabetic LSC-derived Exos by NGS. On average, 24.4 million reads were obtained per sample and the average reads for the vast majority of the data had Q score greater than Q30. Mapping of the sequencing reads aligned to the abundant sequences such as poly A, ribosomal RNA and the mitochondrial chromosomes, as well as to the reference genome including fragments of mRNA and lncRNA transcripts, to small RNA database, and to miRBase. A total of 772 and 337 small RNAs including piRNA, snoRNA and Y_RNA were identified in all samples with the average threshold of more than one Taq Per Million (TPM) > 1 and TPM > 10, respectively (Supplementary Dataset S1).

The number of known miRNAs was calculated after mapping the data and counting relevant entries in miRBase 20. A total of 297 and 150 known miRNAs were identified in all samples with the average threshold of more than one Taq Per Million (TPM) > 1 and TPM > 10 respectively (Supplementary Dataset S2).

The IsomiR analysis was performed for each sample based on the occurrence of count variants for each detected miRNA. These variants were identified by changes in start or stop position, or occurrence of mutations within the read. IsomiRs were not included in differential expression analysis.

**Figure 7:**
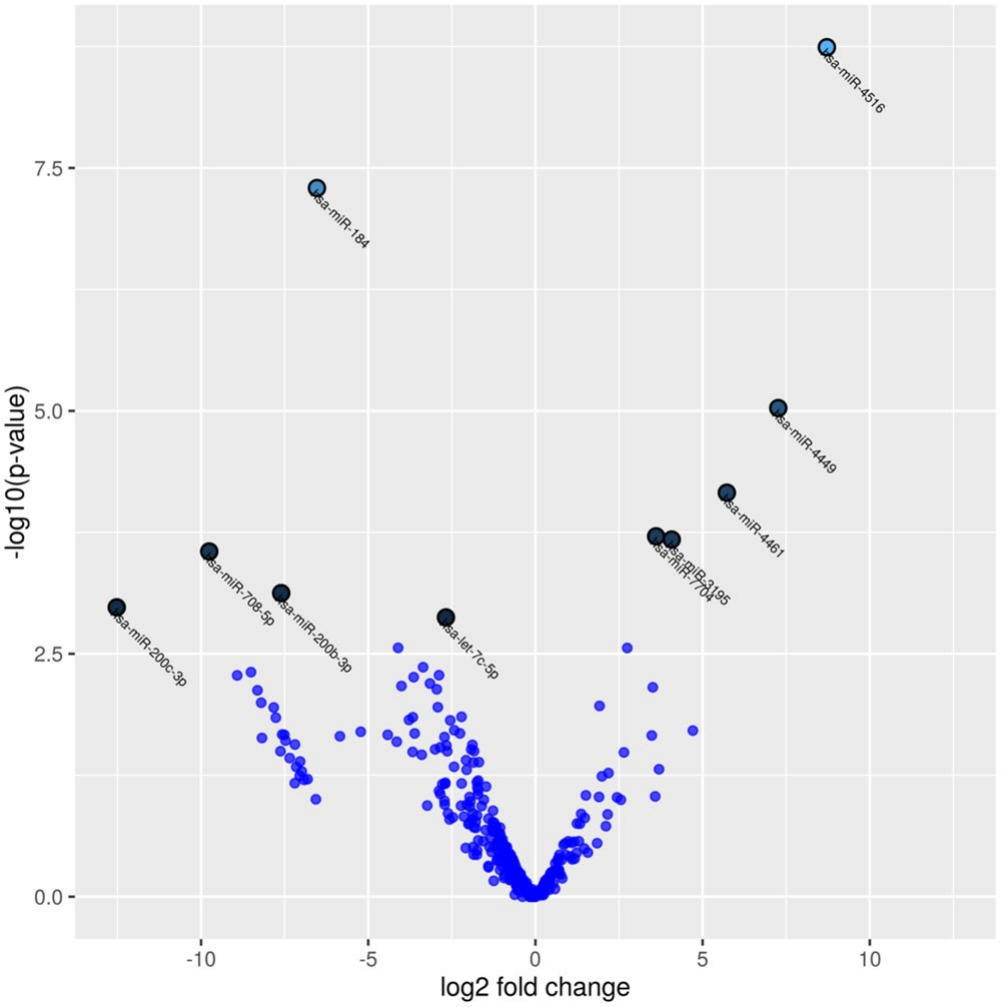
Volcano plot displaying differentially expressed miRNAs between normal and diabetic exosomes. The Y-axis corresponds to the mean expression value of log 10 (p-value), and the X-axis displays the log2 fold change, N-Exos *vs*. DM-Exos.

A set of 312 small RNAs including miRNAs was identified as differentially expressed in DM-Exos vs. N-Exos with the raw p < 0.05 and fold change of greater than 2 (Supplementary Dataset S3). Of these 312 small RNAs and miRNAs 219 showed a false discovery rate (FDR)-adjusted p < 0.05 (Supplementary Dataset S3). miRNA analysis revealed 66 (54 upregulated and 12 downregulated) and 10 (5 upregulated and 5 downregulated) differentially expressed miRNA in DM-Exos vs. N-Exos with the raw p < 0.05 and FDR < 0.05 respectively. Table 1 shows the 20 most differentially expressed miRNAs, and a full list of differentially expressed miRNAs is given in the Supplementary Dataset S4. The Volcano plot shows a quick visual identification of miRNAs with large-magnitude changes, which are also statistically significant (Fig. 7). The plot is constructed by plotting the p-value (−log10) on the y-axis, and the expression fold change between the two experimental groups (N-Exos *vs*. DM-Exos) on the x-axis. The top of the plot (high statistical significance) and the extreme left or right (strongly down- and up-regulated respectively) are the two regions of interest (Fig. 7).

**Table 1:** List of the 20 most significantly differentially expressed microRNAs and annotation.

Names	Log FC	Log CPM	P Value	FDR	Diabetic	Normal
has-miR-4516	−8.716	6.458	1.81E-09	6.02E-07	0.92	216.8
has-miR-184	6.534	15.435	5.08E-08	8.47E-06	76887.86	827.6
has-miR-4449	−7.263	4.216	9.35E-06	0.001037	0.23	52.23
has-miR-4461	−5.725	3.523	6.95E-05	0.005783	0.5	32.87
has-miR-7704	−3.610	5.939	0.000195	0.011698	11.4	135.75
has-miR-3195	−4.076	4.882	0.000211	0.0698	4.15	71.59
has-miR-708-5p	9.756	5.546	0.00028	0.013308	70.76	0
has-miR-200b-3p	7.604	8.513	0.00075	0.031223	622.97	2.25
has-miR-200c-3p	12.523	8.152	0.001051	0.038903	483.32	0
has-let-7c-5p	2.676	12.326	0.001335	0.044449	8032.43	1257.32
has-miR-23b-3p	4.107	8.846	0.002746	0.076447	757.39	46.62
has-miR-146a-5p	−2.747	14.633	0.002755	0.076447	7372.85	49485.89
has-miR-103a-3p	3.359	9.607	0.004341	0.107733	1258.86	125.21
has-miR-1304-3p	8.507	4.447	0.004885	0.107733	30.04	0
has-miR-99a-5p	2.882	14.769	0.005247	0.107733	44332.22	6011.66
has-miR-142-5p	8.507	4.803	0.005273	0.107733	39.82	0
has-miR-500a-3p	3.641	4.945	0.0055	0.107733	42.81	2.25
has-miR-27b-5p	3.154	5.813	0.006391	0.113713	82.84	6.75
has-miR-107	4.005	5.472	0.006785	0.113713	64.51	5.18
has-miR-655-3p	−3.512	2.974	0.006996	0.113713	1.84	25.22
has-miR-125b-3p	2.948	7.837	0.007332	0.113713	352.81	48.87

Log fold change (log FC) between diabetic and normal Exos, raw p-values, Benjamini-Hochberg FDR corrected p-values as well as the average TMM values per group.

### Gene ontology enrichment and pathway analyses

Gene ontology (GO) enrichment analysis was performed to identify GO terms that are significantly associated with differentially expressed miRNAs in DM-Exos *vs*. N-Exos identified to their target genes. Using miRSearch, we mapped the differentially expressed miRNAs (Supplementary Dataset S4) to their target genes and investigated whether specific GO terms were associated with these miRNAs. First, a standard Fisher’s test was used to investigate enrichment of terms between the two test groups. Second, the Elim method was used for more conservative approach by incorporating the topology of the GO network to compensate for local dependencies between GO, which can mask significant GO terms. Comparisons of the predictions from these two methods can highlight truly relevant GO terms. The top 20 most significant GO terms for the target genes found to be differentially expressed in DM- vs. N-Exos and their corresponding annotation for Biological process (BP) are shown in Table 2. Complete GO enrichment analysis of the comparisons of related terms, Molecular functions (MF) and Cellular components (CC) analyses in addition for BP is presented in Supplementary Dataset S5.

**Table 2:** List of the top 20 most significant GO terms for the target genes found to be differentially expressed in DM-Exos vs. N-Exos and their corresponding annotation for Biological process (BP).

GO ID	Term	P value
GO: 0045471	Response to ethanol	5.30-E-05
GO: 0042593	Glucose homeostasis	0.00016
GO: 0045616	Regulation of keratinocyte differentiation	0.00018
GO: 0051150	Regulation of smooth muscle cell differentiation	0.00025
GO: 0060017	Parathyroid gland development	0.00048
GO: 0007219	Notch signaling pathway	0.00054
GO: 0060560	Developmental growth involved in morphogenesis	0.00056
GO: 0043406	Positive regulation of MAP kinase activity	0.00073
GO: 0007157	Heterophilic cell-cell adhesion via plasma membrane cell adhesion molecules	0.00075
GO: 0000083	Regulation of transcription involved in G1/S transition of mitotic cell cycle	0.00096
GO: 0051897	Positive regulation of protein kinase B signaling	0.00098
GO: 0030261	Chromosome condensation	0.00121
GO: 0001843	Neural tube closure	0.00124
GO: 0021846	Cell proliferation in forebrain	0.00138
GO: 0050680	Negative regulation of epithelial cell proliferation	0.00149
GO: 0005979	Regulation of glycogen biosynthetic process	0.00151
GO: 0001755	Neural crest cell migration	0.00154
GO: 0045598	Regulation of fat cell differentiation	0.0017
GO: 0000077	DNA damage checkpoint	0.00176
GO: 0050714	Positive regulation of protein secretion	0.00177

## Discussion

Recent studies have shown that, beside cell-cell contact and soluble factors, EVs constitute an important mechanism for cell-cell communication both in physiological and pathological conditions^11,24^. Further, EVs play an important role in different stem cell niches such as the mesenchymal^25^, cancer^26^, cardiac^27^, neurogenic^28,29^ and bone marrow niches^30^. However, their roles in adult limbal niche remain to be elucidated.

The close spatial arrangement and known communication between putative LESC niche structures and stromal keratocytes^31^ may suggest that EVs may have a role in LEC-LSC crosstalk. There are few studies on corneal EVs/Exos^21,32^ and none in stem cell-enriched limbal area and in disease state such as corneal diabetes. In the present study, we isolated and characterized EV sub-population, exosomes, derived from both normal and diabetic human limbal stromal keratocytes. As confirmed by several analytic approaches, they were in the 50–200 nm size range and positive for CD63, CD81 and HSP70 (exosome markers) suggesting that the majority of our isolated EVs were exosomes. To examine the possible role of Exos in LSC-LEC communications, we showed that exogenous Dil-labeled Exos were uptaken by HCEC and primary LECs *in vitro* and limbal region of *ex vivo* organ-cultured corneas (Fig. 2). Additionally, endogenous Exos released from Dil-labeled LSC were uptaken by primary LECs in co-culture system, suggesting that Exos are involved in paracrine activity of LSC and LEC in limbal niche.

Recent studies have revealed that Exos can affect many biological processes such as cell proliferation, differentiation, angiogenesis, cell migration and wound healing, through their cargo transfer from the originating cells to the recipient cells^33–35^. In order to confirm the role of Exos in LEC-LSC crosstalk in limbal niche, we performed functional analysis of Exos derived from LSC on their recipient cells, LEC. Our study demonstrated that epithelial healing was significantly promoted in wounded primary LEC when incubated with N-Exos compared to untreated wounded cells. However, LEC treated with DM-Exos didn’t show any significant changes in wound healing rate compared to control, untreated wounded cells. Similar studies have previously explored the role of Exos in cell repair and wound healing in other cell types such as in skin^20,33,36,37^, skeletal^38^ and cardiac^39,40^ muscle. A very recent study has shown that Exos derived from human amniotic epithelial cells promoted wound healing and inhibited scar formation in skin^36^. Similarly, a study by Zieske’s group has documented the communication between epithelial cells and keratocytes as well as endothelial cells by Exos secreted by mouse corneal epithelial cells *in vitro* that may suggest their involvement in corneal wound healing^21^.

Our study has also shown that proliferation rate is significantly enhanced in primary LEC when incubated with N-Exos compared to untreated cells. Interestingly, LEC treated with DM-Exos showed less or not any significant changes in proliferation compared to control cells. These data show greater potential of normal Exos in stimulating cell proliferation and wound healing than diabetic Exos. This may suggest that there is a difference in exosomes’ cargos derived from normal and diabetic LSC, which might contribute to the disease state. Furthermore, we observed upregulation of wound healing-related signaling molecule, p-Akt, in wounded LEC and organ-cultured corneas treated with N-Exos compared to untreated wounded cells and DM-Exo treated fellow corneas, respectively. It may be suggested that N-Exos cargos may contain signaling molecules such as p-Akt or its upstream signaling molecules and/or specific miRNAs^35,41,42^ that regulate signaling pathways in their wounded target cells and could actively regulate migration and proliferation in recipient LEC. Additionally, the HSP70 expression in exosomes may promote cell motility beside its other roles such as assisting in proper folding and preventing the aggregation of proteins^43^.

In our study, we assessed for the first time the role of Exos in LESC survival and maintenance. Immunostaining of LEC treated with N-Exos showed upregulation of putative LESC marker, K15, in comparison to untreated cultured cells or DM-Exo treated cells. In addition, organ-cultured corneas treated with N-Exos showed upregulation of putative LESC markers, K15 and FZ7, compared to the organ-cultured fellow corneas treated with DM-Exos. These data further suggest that LSC-derived Exos may contribute to LSC-LEC crosstalk and maintenance of LESC. Downregulation of both K15 and FZ7 by DM-Exos suggest an important difference in exosome cargos derived from normal and diabetic LSC contributing to the disease state. These data are in line with our previous results on downregulation of a number of putative LESC markers in human diabetic corneas^44^. In fact, the mechanism of this effect may be related to exosomes secreted by diabetic LSC that may not support normal LESC maintenance.

In all types of EVs including exosomes, miRNAs have been found in large amounts, which may exert various effects in recipient cells due to their key regulatory roles in gene expression^45^. Thus, we performed comparative exosomal small RNA profiling using NGS analysis (Exiqon) for both normal and DM LSC-derived Exos, which could help us reveal the mechanism of exosomal function in normal and diabetic limbal niche. We quantitatively identified the spectrum of small RNAs (including miRNAs) profiles of N and DM LSC-derived Exos and those miRNAs that abnormally expressed in DM-Exos of diabetic corneas (Supplementary Dataset S3). The top differentially expressed miRNA, miR-4516, has been shown to inhibit skin keratinocyte migration by targeting fibronectin/integrin α9 signaling^46^. Paradoxically, it was significantly downregulated in DM-Exos although diabetes induces corneal wound healing impairment. As fibronectin is not significantly changed in diabetic corneas, miR-4516 in the cornea may work on other targets, e.g., on MMP-2 that is elevated in the diabetic corneas, presumably in the corneal stroma^47^. Alternatively, downregulation of miR-4516, may present an attempt of the diabetic stromal cells to preserve normal wound healing, which may be eventually counteracted by other miRs that become elevated in diabetes, for instance, miR-146a that inhibits wound healing-stimulating EGFR^48^. Interestingly, miR-184, the most abundantly expressed miRNA in central corneal epithelium^49,50^ is among the top differentially expressed miRNAs and is significantly upregulated in DM-Exos. Its key role in corneal epithelial homeostasis has been shown in several studies^50–52^. It has been suggested to function in corneal angiogenesis by targeting VEGF and Akt signaling^50^, regulate the transition from proliferation to early differentiation^52^, while its abnormal expression or mutation resulted in impaired homeostasis leading to corneal diseases such as familial severe keratoconus^51^. Therefore, its upregulation in DM-Exos may lead to the alteration of limbal niche homeostasis resulting in diabetic disease state such as altered proliferation and differentiation^53,54^. Both miR-200b-3p and miR-200c-3p, which are significantly upregulated in DM-Exos, have been shown to inhibit growth and motility^55,56^, which may contribute to slow wound healing and migration of the diabetic epithelium. Interestingly, miR-146a is another differentially expressed miRNA in DM *vs*. normal LSC-Exos with the potential of targeting and downregulating signaling molecules and putative stem cell markers, K15 and FZ7, as we have shown previously^57^.

We also identified the spectrum of small RNAs including, piRNA, tRNA, snRNA and Y_RNAs, in N and DM LSC-derived Exos, and the abnormally expressed small RNAs in DM-Exos. These categories of small RNAs are involved with both epigenetic and post-transcriptional gene silencing, splicing and DNA replication. Further studies are required to determine the functions of the differentially expressed small RNAs including miRNAs in limbal niche.

To identify differentially expressed miRNA target genes that might be responsible for the diabetic corneal abnormalities, gene ontology (GO) and pathway analysis were performed to determine the underlying mechanisms. Biological function, cellular component, and molecular function were the most related terms in the GO analysis. Interestingly, the most significant GO terms include insulin receptor signaling, cell cycle regulators, signaling molecules, and negative regulation of epithelial cell proliferation, which have been shown to be dysregulated in diabetes^54^. In addition, significant GO terms such as Notch signaling pathway and regulation of keratinocyte differentiation of differentially expressed miRNA target genes suggest the alteration of LESC function observed in diabetic cornea^54^. These data suggest a disturbed LSC-LEC communication in the diabetic cornea, which may lead to disease alterations.

In conclusion, this is the first study showing the Exos’ role in limbal niche in LSC-LEC communications in healthy and diabetic corneas. We have documented Exos’ influence on a key signaling molecule involved in migration and proliferation and putative LESC marker in *in vitro* and *ex-vivo* organ-cultured corneas. Further, we identified both normal and DM LSC-derived Exos’ cargos and differentially expressed small RNAs including miRNAs in DM-Exos, which may have roles in disease state. Further studies are required to determine their functions in normal and diabetic cornea. The presented data may contribute to better understanding of the complexity of the limbal niche in physiological and pathological conditions. This would help us develop more effective therapeutic approaches by targeting the niche and its cellular components for the treatment of corneal diseases such as diabetic keratopathy.

## Materials and Methods

### Human specimens

Age-matched human autopsy healthy and diabetic corneas (Supplementary Table S1) were received from National Disease Research Interchange (NDRI, Philadelphia, PA) in Optisol storage medium; donor identity was withheld by the supplier. NDRI has a human tissue collection protocol approved by a managerial committee and subject to National Institutes of Health oversight. In all cases the required informed consent from donors next of kin specifying the use of postmortem tissue for research was obtained by NDRI-affiliated eye banks. The work reported here was covered by approved Cedars-Sinai Medical Center IRB protocols EX-1055 and Pro00019393. Corneas were harvested within 5 hours of donor death and reached our laboratory within 24 h of death, and studies were conducted in accordance with approved guidelines.

### Isolation and maintenance of primary limbal epithelial and stromal cells

Primary limbal epithelial and stromal cells were dissociated from the age-matched autopsy normal and diabetic limbal rims. LEC containing LESC were isolated from corneoscleral rims by Dispase/Trypsin digestion to dissociate LEC from stroma, and were maintained and characterized as previously described^49,57^. After removal of epithelial cells by enzymatic digestion, corneal stroma was chopped and kept in 1 mg/ml collagenase type IV solution at 37 °C overnight^9^. LSC were filtered through a 70 μm  mesh, washed and re-suspended in complete culture medium (CCM) [DMEM/F12 supplemented with B27, N2, 1% antibiotic/antimycotic and 5 ng/ml basic fibroblast growth factor (FGF2), PeproTech Inc, Rocky Hills, NJ], plated at 8 × 10^3^ cells/cm^2^ and kept in the incubator at 37 °C and 5% CO2. Cells were passaged after 70–80% confluence using TrypLE express (Thermo Fisher Scientific, Waltham, MA).

### Maintenance of human organ-cultured cornea

Corneal organ cultures were established as described in details^58^ and were maintained in Dulbecco’s Modified Eagle’s Medium (Thermo Fisher Scientific) with 1X insulin-transferrin-selenite (Sigma-Aldrich), 1X non-essential amino acids, and 1X antibiotic/antimycotic mix (Thermo Fisher Scientific).

### Isolation of EVs/Exos from primary LSC culture supernatants

Normal and diabetic LSC were cultured in serum free CCM. EVs were prepared from conditioned media of normal and diabetic LSC using ultracentrifugation and ExoQuick-TC (SBI, Palo Alto, CA) precipitation as described previously^59^. In brief, an initial spin was performed at 10,000 × g at room temperature for 10 min for each sample to remove cells and debris, followed by filtration through 0.2 µm filter to remove cell debris and particles larger than 200 nm. The resulting cell free medium was concentrated by ultrafiltration using Amicon® Ultra-15 membrane with molecular weight cutoff of 3,000 Daltons (Millipore, St. Louis, MO). EVs were precipitated in Exo-Quick-TC (SBI), exosome isolation reagent, following the manufacturer’s protocol. Briefly, concentrated cell culture supernatant and Exo-Quick-TC reagent were added proportional to the starting sample volume, mixed and incubated at 4 °C for up to an hour and then centrifuged at 10,000 × g for 30 minutes at room temperature to precipitate Exo pellets. The Exo pellet was re-suspended in appropriate buffer and stored at −80 °C immediately after isolation until further analysis.

### Transmission electron microscopy (TEM)

Isolated Exos (1 µg) were resuspended in phosphate-buffered saline (PBS) and the suspension was adsorbed on carbon-formvar 300 mesh grids for 30 min, fixed with 2% glutaraldehyde, washed and stained with 2% uranyl acetate (UA). The grids were dragged on a piece of filter paper to remove the excess of UA, allowed to dry and examined on a JEOL 100CX electron microscope at 60 kV. Images were collected on type 4489 EM film, and the negatives scanned to create digital files.

### NanoSight particle size analysis by dynamic light scattering

Exos were suspended in PBS and analyzed in real time using dynamic light scattering measurements with NanoSight LM10-HS instrument equipped with a laser (638 nm) and Nanoparticle Tracking Analysis software version 2.3, Build 0033 (NanoSight, Westborough, MA). Post-acquisition settings were based on the manufacturer’s recommendations and kept constant between the samples. Each video was analyzed to obtain particle size distribution profiles and concentration measurements.

### Flow cytometry

Flow cytometry was performed as per manufacturer’s instructions. Briefly, the harvested and fixed keratocytes from passage 2–3 (1.0 × 10^6^ cells/ml) were incubated with unconjugated primary antibodies (Supplementary Table S2), rabbit anti-lumican (aa64–91) and mouse anti-ALDH3 (1B6) followed by incubating with secondary antibodies (Li-Cor Biosciences, Lincoln, NE) and measured with a flow cytometer BD LSR II instrument (BD Biosciences, San Jose, CA). Pooled Exos from keratocytes (1 × 10^8^ per 10 µl of Dynabeads®) were incubated overnight at 4 °C with Dynabeads® magnetic beads (Invitrogen, Carlsbad, CA) coated with primary monoclonal antibody specific for the CD63 (Supplementary Table S2) membrane antigen. To detect the presence of specific antigens like CD81 or CD63, the exosome-coated beads were incubated with PE-CD63 (H5C6) and APC-CD81(5A6) antibodies (Supplementary Table S2) for 45 min at RT on a sample shaker (1000 rpm) followed by isolation buffer (PBS with 0.1% BSA, filtered through 0.2 µm filter) washes. Complexes were resuspended in isolation buffer and subjected to flow cytometry using BD LSR II instrument (BD Biosciences), where at least 50,000 events were collected, and results were analyzed by Flowjo software.

### Western blot analysis and immunostaining

Western blot was performed as described previously^48^. Briefly, treated cells or Exos were lysed and suspended in Tris-glycine sample buffer with proteinase inhibitor EDTA-free cocktail. Equal amounts of lysates were subjected to SDS-PAGE and transferred onto nitrocellulose membranes. Blots were blocked followed by incubation with primary antibodies (Supplementary Table S2) overnight at 4 °C. IRDye LiCor secondary antibodies (Li-Cor Biosciences) were used for protein detection with LiCor Odyssey CLX imaging system (Li-Cor Biosciences). Quantification of protein bands was done with Image Studio. Immunostaining was performed as described previously^57^. Isotype control (IgG2a kappa) was used as a negative control for K15 antibody.

### Exos cellular uptake

Cellular uptake of Exos was followed using confocal microcopy. LSC- derived Exos were labeled with Dil fluorescent dye (1,1′-dioctadecyl-3,3,3′,3′-tetramethylindocarbocyanine perchlorate; Thermo Fisher Scientific) that labels the plasma membrane, according to the manufacturer’s instructions. Briefly, Exos were incubated with Dil dye for 1 h at RT in the dark followed by two washes in PBS. Next, 10 μg/ml and/or 25 μg/ml Dil-labeled Exos in a total volume of 100 μl were diluted in respective medium as described previously^36^ and were added to N or DM (N/DM) LEC cultures or organ-cultured corneas for 24–48 h and washed prior to staining with 10 μM calcein-AM (Thermo Fisher Scientific) at 37 °C for 30 min in the dark. Cells or organ-cultured corneas were briefly washed and examined under a Zeiss LSM-780 confocal microscope (BioSciences, Jena, Germany). Control group was cultured in respective medium with added 100 μl of PBS.

### Co-culture assay

Dil fluorescent dye was added to limbal keratocytes and allowed to incubate at 37 °C for 30 min. Cells were washed and transferred for co-culturing with LEC in 24-well plate with cell culture porous membrane inserts. Dil-labeled keratocytes were seeded onto the 0.4 µm inserts which allow transport of EVs but no cells. After 48 hours the inserts were removed and LECs were labeled with calcein-AM for 30 min at 37 °C and imaged with confocal microscopy.

### *In-vitro* MTS proliferation and wound healing assays

Proliferation assay was performed as described previously^49^. Briefly, HCEC were seeded on 96-well plates at 5,000/well and 25 μg N/DM LSC-derived Exos were added to the basal medium without growth factors. After 24 h, proliferation was measured using MTS assay (CellTiter 96 Aqueous One Solution Cell Proliferation Assay, Promega, Madison, WI). Scratch wound assay was performed either on treated HCEC or LEC with 25 μg N/DM-Exos as described^48^. Briefly, treated LEC or HCEC at confluence were scratch wounded using the pipette tip and photographed at time 0. The wounds were allowed to heal and photographed every 6 h. All images were then analyzed using ImageJ software. The percent area healed was calculated with reference to time 0. Wound healing in organ-cultured corneas was performed as published^57^. A 5 mm wound in central cornea was created using a disk soaked in n-heptanol and incubated with Exos (3.5 × 10^8^) and wound closure was monitored over time.

### Small RNA Next Generation Sequencing (NGS)

#### Exo RNA isolation

Total RNA within Exos was isolated by miRCURY RNA Isolation Kit–Cell & Plant (Exiqon, Woburn, MA) according to the manufacturer’s instructions. The concentration of total RNA was determined by NanoDrop spectrophotometer (Thermo Fisher Scientific) and their quality was analyzed using 2100 Bioanalyzer (Agilent, Santa Clara, CA). NGS was performed by Exiqon (Denmark).

#### Small RNA library preparation

The library preparation was done using the NEBNext® Small RNA Library preparation kit (New England Biolabs). Sequencing libraries were generated by ligation of adapters to the small RNAs extracted from 100 ng of total RNA for each sample followed by reverse transcription and PCR amplification (15 cycles) and purification of small RNA libraries. QC for the generated libraries was performed using either Bioanalyzer 2100 (Agilent, Carpinteria, CA) or TapeStation 4200 (Agilent) and they were size sorted using the LabChip XT (Perkin Elmer, Inc, San Jose, CA) aiming to select the fraction with the size corresponding to microRNA libraries (~145 nt). The library pools were quantified using the qPCR KAPA Library Quantification Kit (KAPA Biosystems, Wilmington, MA).

#### Small RNA and miRNA sequencing

The library pool was sequenced on a NextSeq 500 sequencing instrument according to the manufacturer’s instructions. Raw data were de-multiplexed and FASTQ files for each sample were generated using the bcl2fastq software (Illumina Inc., San Diego, CA). FASTQ data were checked using the FastQC tool^60^. RNA adapters were trimmed off and the resulting reads were mapped to miRBase and small RNA database.

#### Data analysis workflow

Adapters were removed from the sequences using cutadapt^61^. Bowtie2 version 2.2.2^62^ was used to map against the human reference genome (GRCh37). No mismatches were allowed for mapping against miRbase 20, one mismatch in the first 32 bases of the read and no indels were allowed for mapping to the genome.

#### NGS statistical analysis

For the statistical analyses presented in this study, the trimmed mean of M-values normalization method (TMM normalization) was used^63^, in addition to TPM normalization, which compensate for sample specific effects caused by the variation in library size/sequencing depth between samples. The differential expression analysis was done using TMM in the EdgeR statistical software package^64^. For gene ontology enrichment analysis, the TopGO R package^65^, two different statistical tests were used and compared. First, a standard Fisher’s test was used to investigate enrichment of terms between the two test groups. Second, the ‘Elim’ method takes a more conservative approach by incorporating the topology of the GO network to compensate for local dependences between GO, which can mask significant GO terms. Comparisons of the predictions from these two methods can highlight truly relevant GO terms.

### Statistical analysis

Experiments were analyzed by Student’s t-test for two groups, or ANOVA for three or more groups with p < 0.05 considered significant, using Prism6 (GraphPad Software, San Diego, CA).

## Electronic supplementary material

Supplementary Tables

Supplementary Figures

Supplementary Dataset S1

Supplementary Dataset S2

Supplementary Dataset S3

Supplementary Dataset S4

Supplementary Dataset S5

Supplementary Information

## Data Availability

All data generated or analyzed during this study are included in this published article (and its Supplementary Information files).
