# Supplementary material for: Exosomes from normal and diabetic human corneolimbal keratocytes differentially regulate migration, proliferation and marker expression of limbal epithelial cells
Source: Sci Rep. 2018 Oct 11;8:15173. doi: 10.1038/s41598-018-33169-5 (PMC6182003; doi:10.1038/s41598-018-33169-5)
Supplement: Supplementary file 1 — Supplementary Tables [file 41598_2018_33169_MOESM1_ESM.pdf]

Exosomes from normal and diabetic human corneolimbal keratocytes differentially regulate migration, proliferation and marker expression of limbal epithelial cells

Aleksandra Leszczynska[1,2], Mangesh Kulkarni[1,2], Alexander V. Ljubimov[1,2,3], Mehrnoosh Saghizadeh*[1,2,3]

[1]Biomedical Sciences, [2]Regenerative Medicine Institute Eye Program, [3]David Geffen School of Medicine, University of California Los Angeles, Los Angeles, California, USA.

* Correspondence to: Dr. Mehrnoosh Saghizadeh, Eye Program, Regenerative Medicine Institute, Cedars-Sinai Medical Center, 8700 Beverly Boulevard, AHSP-A8109, Los Angeles, CA 90048, USA. Tel. 1-310-248-8696, e-mail ghiamm@cshs.org

Supplementary Table S1. List of corneas used in this study.

Case number	Age	Gender	Cause of death	Type of DM/duration (Years)	History of eye diseases
N15-09	15	M	Cerebrovascular Accident	N/A	None
N15-23	22	M	Electrocution/Cardiac Arrest	N/A	None
N15-24	35	M	Coronary Artery Atherosclerosis	N/A	None
N16-04	71	F	End stage COPD	N/A	Cataracts
N16-13	87	M	Ruptured Aortic Aneurysm	N/A	Cataracts
N16-14	76	F	Cardiac/pulmonary failure	N/A	None
N16-16	23		Traumatic Arrest/Accident	N/A	None
N16-19	84	M	Cardiopulmonary arrest	N/A	Cataracts
N16-23	30	M	Cardiac	N/A	None
N 16-22	58	F	Metastatic Melanoma	N/A	None
N16-29	69	M	Metastatic adenocarcinoma	N/A	Cataracts
N16-30	46	F	Brain aneurysm	N/A	None
N17-11	52	M	Hypertension	N/A	Cataracts
DM14-43	73	F	Congestive heart/renal failure	NIDDM/10	None
DM15-06	65	F	Respiratory Failure	IDDM/unknown	
DM15-22	84	F	Respiratory Failure	NIDDM/15	Cataracts
DM16-27	71	F	Cardiac	NIDDM/15	None
DM16-28	88	F	Cardiac arrest	NIDDM/40	Cataracts
DM17-47	74	F	Anoxic brain injury	NIDDM/17	None

N, normal; DM, diabetic mellitus; IDDM, T1DM; NIDDM, T2DM; M, male; F, female; COPD, chronic obstructive pulmonary disease.

Supplementary Table S2. List of antibodies used in this study

Antigen	Antibody	Source	Assay	Dilution
CD63	Mouse mAb sc-5275	Santa Cruz Biotechnology	WB	1:500
CD63	Mouse mAb 353003 (clone H5C6)/PE	BioLegend	FC	1:100
CD81	Mouse mAb 349509 (clone 5A6)/APC	BioLegend	FC	1:100
HSP70	Mouse mAb sc-24	Santa Cruz Biotechnology	WB	1:500
K12	Goat pAb sc-17098	Santa Cruz Biotechnology	IF	1:30
K15	Mouse mAb sc-47697	Santa Cruz Biotechnology	IF	1:100
K17	Mouse mAb sc-58726	Santa Cruz Biotechnology	IF<br>WB	1:100
β-Actin	Mouse mAb A5316	Sigma-Aldrich	WB	1:6000
β-Actin	Rabbit mAb 8457	Cell Signaling Technology	WB	1:1000
β-Tubulin	Rabbit mAb 2128	Cell Signaling Technology	WB	1:1000
Lumican	Rabbit pAb LS-C100251 (clone aa64-91) unconjugated	LifeSpan BioSciences Inc.	WB<br>FC<br>IF	1:1000<br>1: 50<br>1:100
Keratocan	Rabbit pAb LS-B8216	LifeSpan BioSciences Inc.	IF	1:500
ALDH3	Mouse mAb LS-B14523 (clone 1B6) unconjugated	LifeSpan BioSciences Inc.	IF<br>FC	1:100<br>1:100
α-SMA	Mouse mAb F3777	Sigma-Aldrich	IF	1:300
p-Akt	Rabbit pAb 9271S	Cell Signaling Technology	WB	1:1000
p-p38	Rabbit mAb 9215	Cell Signaling Technology	WB	1:1000
FZ7	Rat mAb MAB1981	R & D Systems Inc	IF	1:40

pAb, polyclonal antibody; mAb, monoclonal antibody. K, keratin; FZ, Frizzled; ALDH3, aldehyde dehydrogenase 3; WB, western blot; FC, flow cytometry; PE, Phycoerythrin (Fluorochrome), APC, Phycobilin pigments (Fluorochrome); IF, immunofluorescence.
