# Supplementary material for: Exosomes from normal and diabetic human corneolimbal keratocytes differentially regulate migration, proliferation and marker expression of limbal epithelial cells
Source: Sci Rep. 2018 Oct 11;8:15173. doi: 10.1038/s41598-018-33169-5 (PMC6182003; doi:10.1038/s41598-018-33169-5)
Supplement: Supplementary file 2 — Supplementary Figures [file 41598_2018_33169_MOESM2_ESM.pdf]

Exosomes from normal and diabetic human corneolimbal keratocytes differentially regulate migration, proliferation and marker expression of limbal epithelial cells

Aleksandra Leszczynska[1,2], Mangesh Kulkarni[1,2], Alexander V. Ljubimov[1,2,3], Mehrnoosh Saghizadeh*[1,2,3]

[1]Biomedical Sciences, [2]Regenerative Medicine Institute Eye Program, [3]David Geffen School of Medicine, University of California Los Angeles, Los Angeles, California, USA.

* Correspondence to: Dr. Mehrnoosh Saghizadeh, Eye Program, Regenerative Medicine Institute, Cedars-Sinai Medical Center, 8700 Beverly Boulevard, AHSP-A8109, Los Angeles, CA 90048, USA. Tel. 1-310-248-8696, e-mail ghiamm@cshs.org

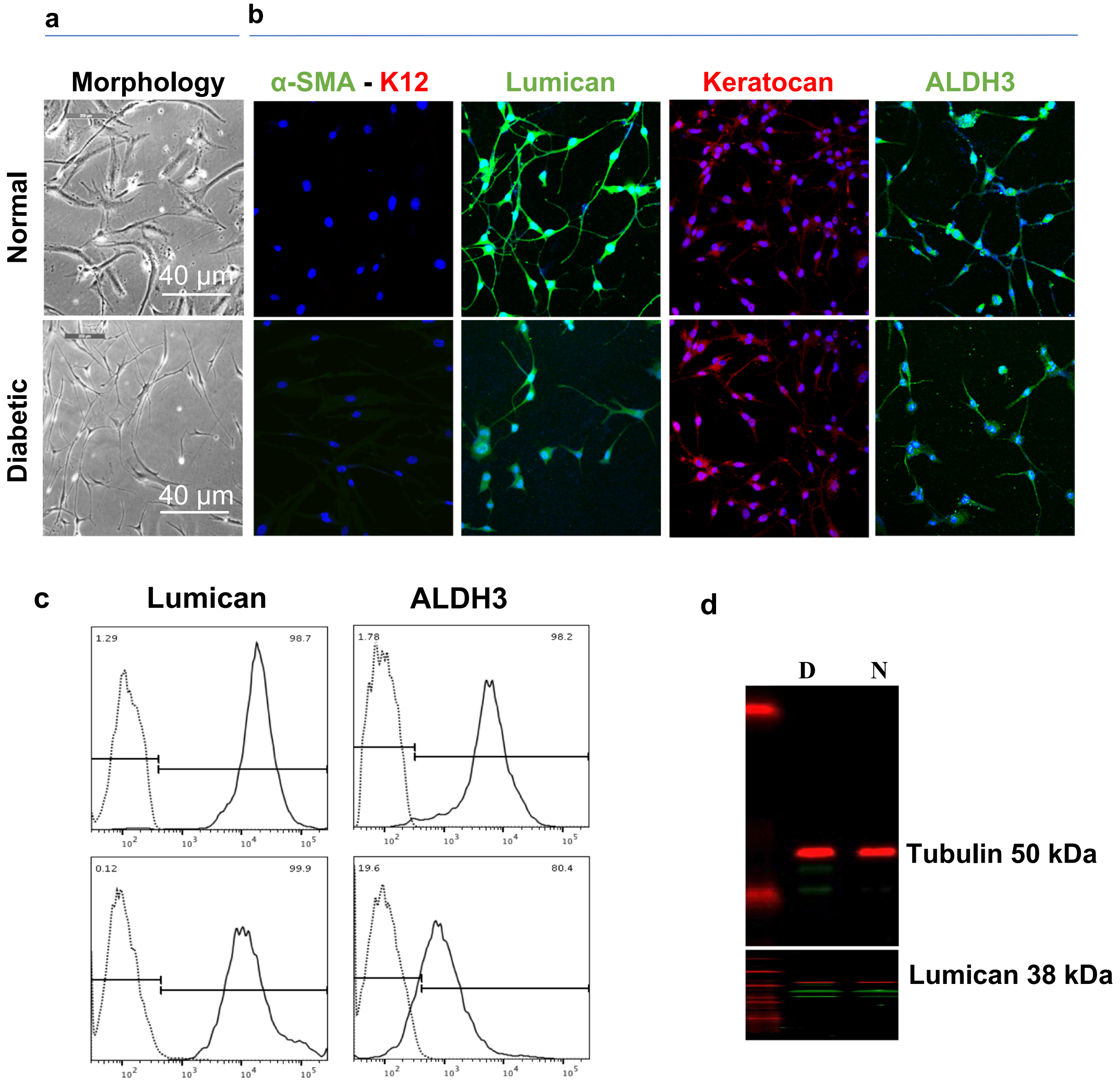

Supplementary Figure S1. Characterization of N and DM human primary cultured LSCs or keratocytes. a. Bright-field light microscopy of limbal stromal cells (P1-4) showed specific keratocyte dendritic morphology. **b.** LSC expressed lumican, keratocan and ALDH3 but not α-SMA or K12. DAPI was used as a nuclear stain. **c.** Flow cytometry further confirmed the expression of lumican and ALDH3 by LSC. The doted line represents background staining obtained with isotype-matched control antibody. **d.** Western blot analysis of total extracted protein showed lumican expression in both normal and DM primary LSC.

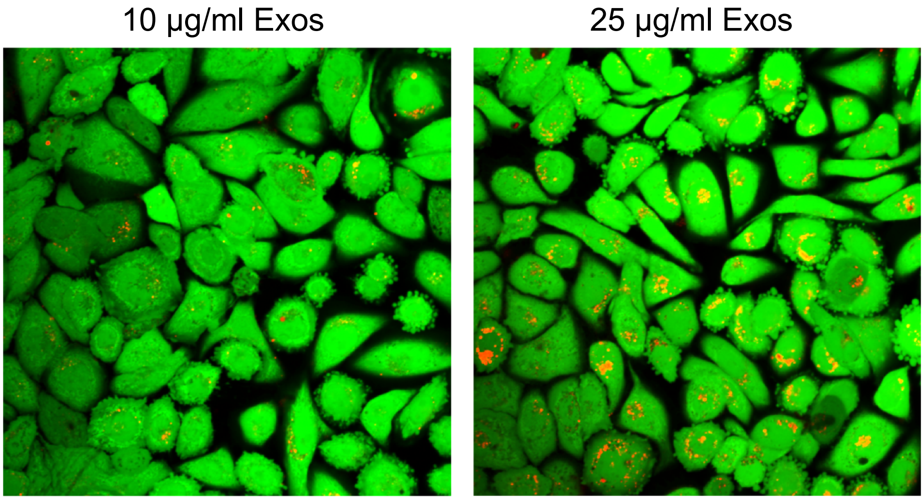

Supplementary Figure S2. **Dil-labeled normal human LSC-derived EVs can be internalized by human primary LEC.** 10 and 25 µg/ml Dil-labeled LSC-derived Exos were incubated (direct addition of Dil-labeled Exos) with LECs for 24h. Exos were internalized by most of the cultured cells at the concentration of 25 µg/ml.

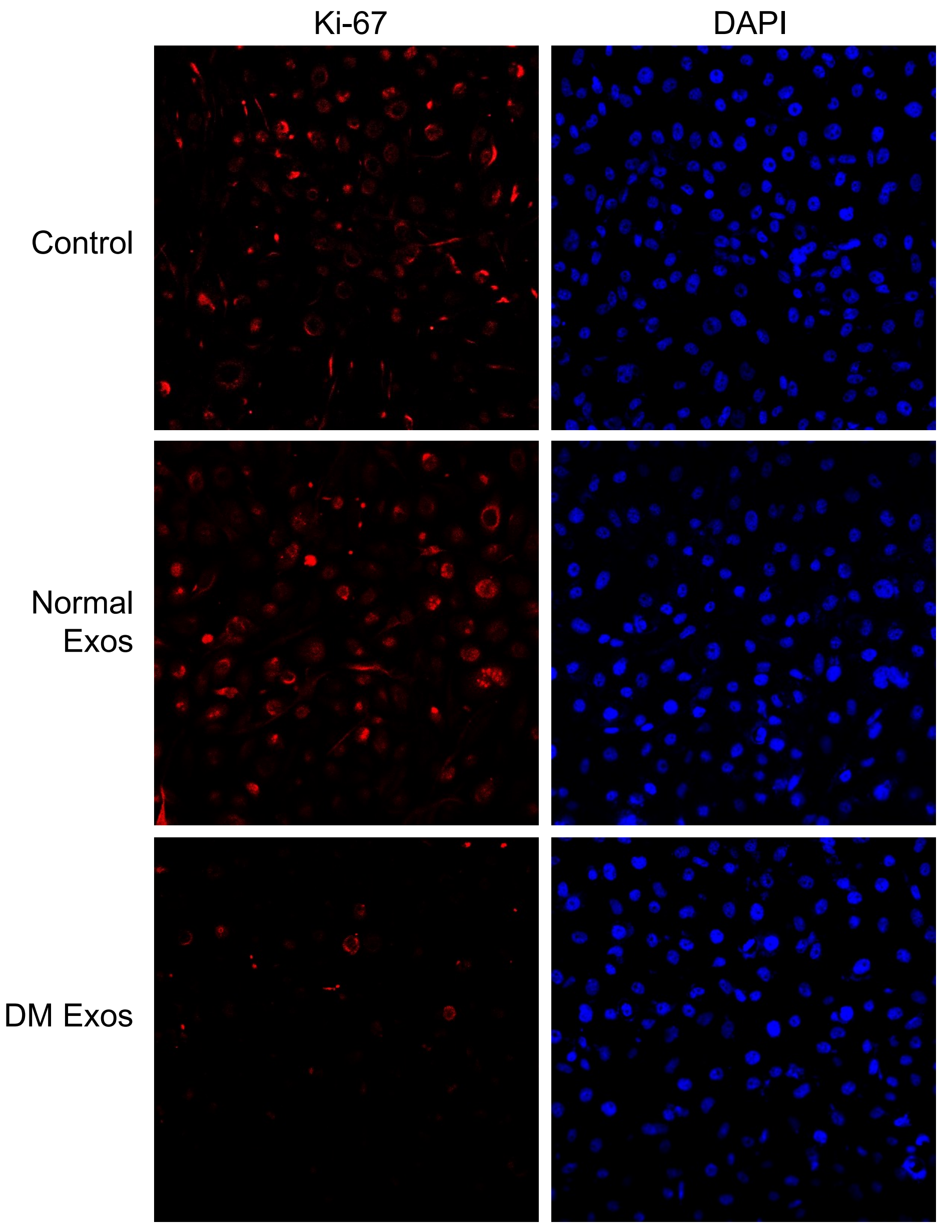

Supplementary Figure S3. Normal EVs increase proliferation of normal primary LECs. N-Exo treatment in normal primary LECs led to increased expression of Ki-67 compared to those treated with DM-Exos or untreated control cells. While, DM-Exo treatments decreased the expression of Ki-67 compared to untreated control cells.

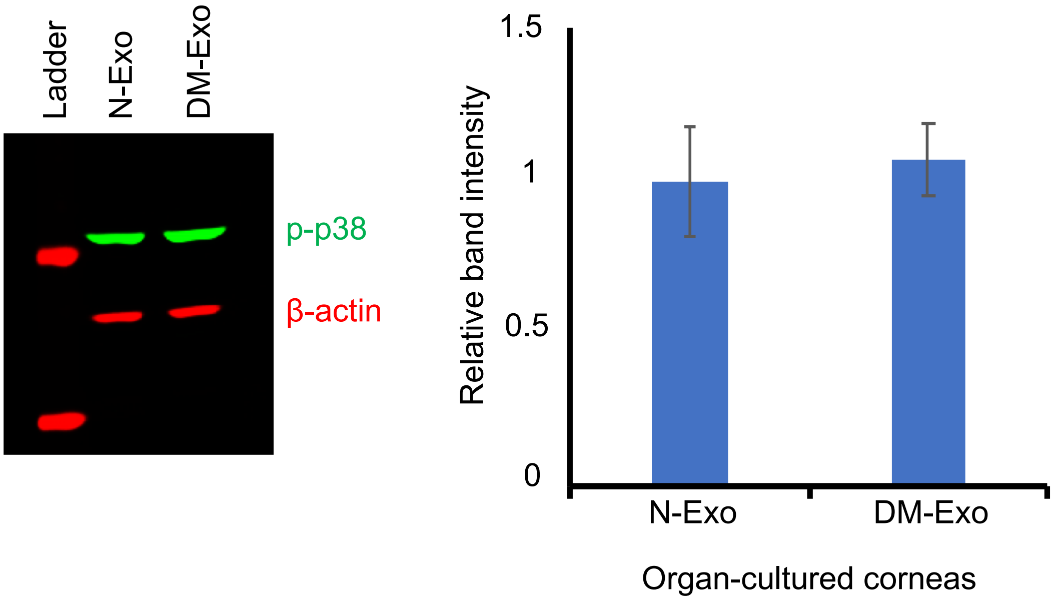

Supplementary Figure S4. Western blot analysis showed no significant changes in p-p38 expression in wounded organ-cultured corneas treated with N-Exos compared to the fellow corneas treated with DM-Exos. Antibody to β-actin was used as equal loading control and for semi-quantitation.
