# Supplementary material for: Exosomes from normal and diabetic human corneolimbal keratocytes differentially regulate migration, proliferation and marker expression of limbal epithelial cells
Source: Sci Rep. 2018 Oct 11;8:15173. doi: 10.1038/s41598-018-33169-5 (PMC6182003; doi:10.1038/s41598-018-33169-5)
Supplement: Supplementary file 8 — Supplementary Information [file 41598_2018_33169_MOESM8_ESM.pdf]

Exosomes from normal and diabetic human corneolimbal keratocytes differentially regulate migration, proliferation and marker expression of limbal epithelial cells

Aleksandra Leszczynska[1,2], Mangesh Kulkarni[1,2], Alexander V. Ljubimov[1,2,3], Mehrnoosh Saghizadeh*[1,2,3]

[1]Biomedical Sciences, [2]Regenerative Medicine Institute Eye Program, [3]David Geffen School of Medicine, University of California Los Angeles, Los Angeles, California, USA.

* Correspondence to: Dr. Mehrnoosh Saghizadeh, Eye Program, Regenerative Medicine Institute, Cedars-Sinai Medical Center, 8700 Beverly Boulevard, AHSP-A8109, Los Angeles, CA 90048, USA. Tel. 1-310-248-8696, e-mail ghiamm@cshs.org

a

b

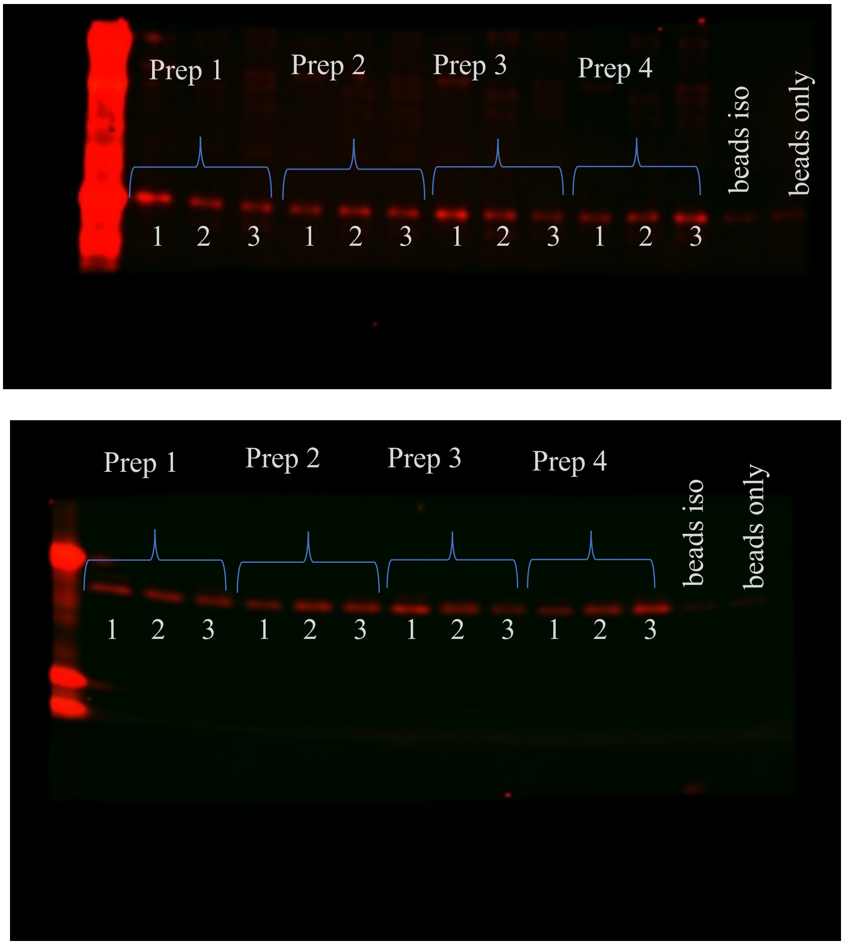

HSP70 70kDa

CD63 30-60kDa

Figure 1d. Characterization of normal and DM human LSC-derived Exos. Full length gels and western blots showed expression of typical exosomal markers HSP70 (a) and CD63 (b) in both N and DM vesicles for four replicates.

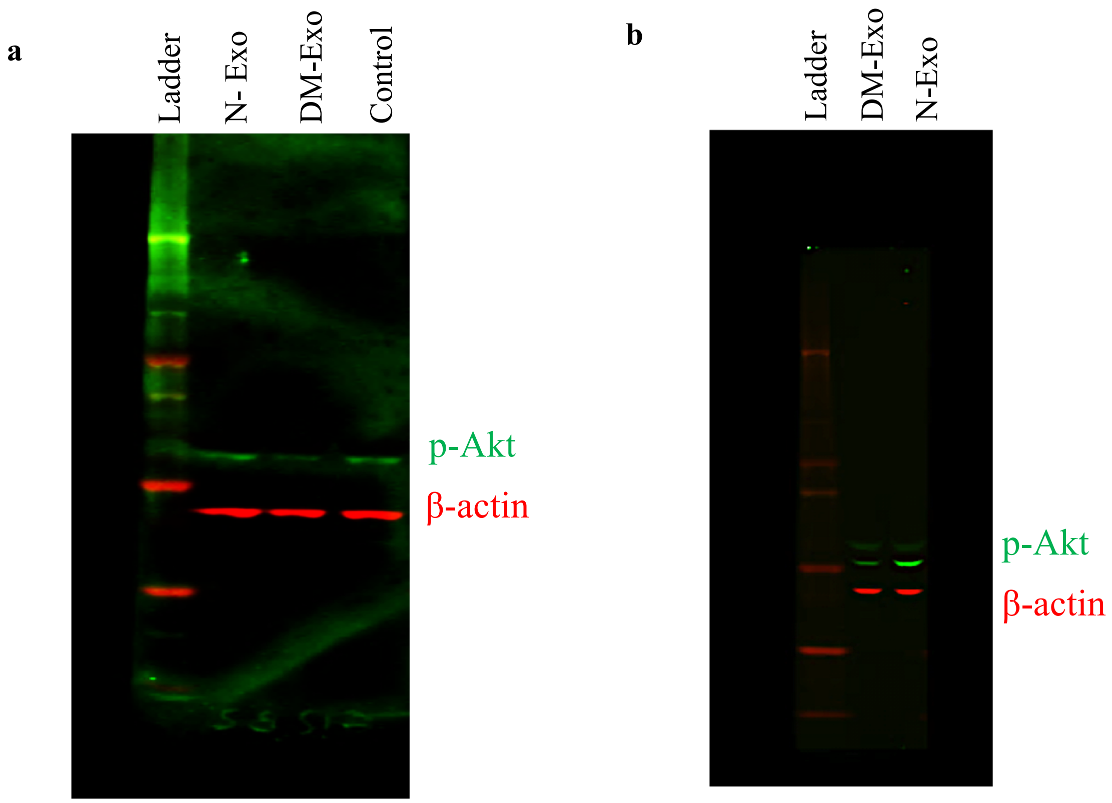

Figure 4. Full gels of western blot analysis of p-Akt expression in wounded LEC and organ-cultured corneas treated with normal or DM LSC-derived Exos. **a.** Total extracted protein from wounded primary LEC treated with N/DM-DM Exos and untreated cells (control) was separated on gradient SDS-PAGE gels, transferred to nitrocellulose and probed with antibodies to p-Akt. Full length gels and western blots showed normal-Exo treatment increases protein levels of p-Akt *vs.* control (PBS/untreated) and DM-Exo treated cells. **b.** Full length gels and western blots showed increases p-Akt expression in wounded organ-cultured corneas treated with N-Exos compared to the fellow corneas treated with DM-Exos, Antibody to β-actin was used as equal loading control and for semi-quantitation. * $p<0.05$ by paired two-tailed t test.

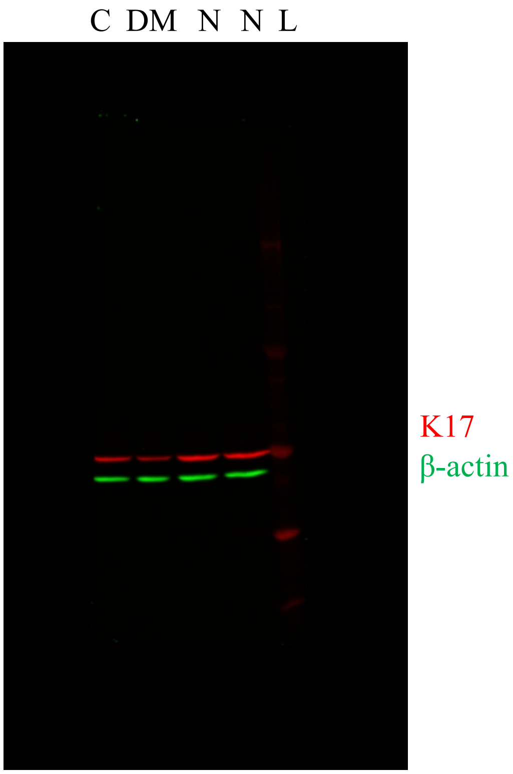

Figure 6b. Effect of N- or DM-Exos on LESC marker expression in normal organ-cultured corneas and primary LECs. Full length gel and western blot shows that N-Exo treatment increased, whereas DM-Exo treatment decreased K17 protein expression level in primary LECs compared to control treated cells, which did not reach significance. Antibody to β-actin was used as equal loading control and for semi-quantitation.
